# Pyoverdine, the Major Siderophore in *Pseudomonas aeruginosa*, Evades NGAL Recognition

**DOI:** 10.1155/2012/843509

**Published:** 2012-09-02

**Authors:** Mary E. Peek, Abhinav Bhatnagar, Nael A. McCarty, Susu M. Zughaier

**Affiliations:** ^1^School of Chemistry & Biochemistry, Georgia Institute of Technology, Atlanta, GA 30332, USA; ^2^Division of Pulmonology, Allergy/Immunology, Cystic Fibrosis, and Sleep, Department of Pediatrics and Emory+Children's Center for Cystic Fibrosis Research, Emory University School of Medicine and Children's Healthcare of Atlanta, Atlanta, GA 30322, USA

## Abstract

*Pseudomonas aeruginosa* is the most common pathogen that persists in the cystic fibrosis lungs. Bacteria such as *P. aeruginosa* secrete siderophores (iron-chelating molecules) and the host limits bacterial growth by producing neutrophil-gelatinase-associated lipocalin (NGAL) that specifically scavenges bacterial siderophores, therefore preventing bacteria from establishing infection. *P. aeruginosa* produces a major siderophore known as pyoverdine, found to be important for bacterial virulence and biofilm development. We report that pyoverdine did not bind to NGAL, as measured by tryptophan fluorescence quenching, while enterobactin bound to NGAL effectively causing a strong response. The experimental data indicate that pyoverdine evades NGAL recognition. We then employed a molecular modeling approach to simulate the binding of pyoverdine to human NGAL using NGAL's published crystal structures. The docking of pyoverdine to NGAL predicted nine different docking positions; however, neither apo- nor ferric forms of pyoverdine docked into the ligand-binding site in the calyx of NGAL where siderophores are known to bind. The molecular modeling results offer structural support that pyoverdine does not bind to NGAL, confirming the results obtained in the tryptophan quenching assay. The data suggest that pyoverdine is a stealth siderophore that evades NGAL recognition allowing *P. aeruginosa* to establish chronic infections in CF lungs.

## 1. Introduction

**Figure 1:**
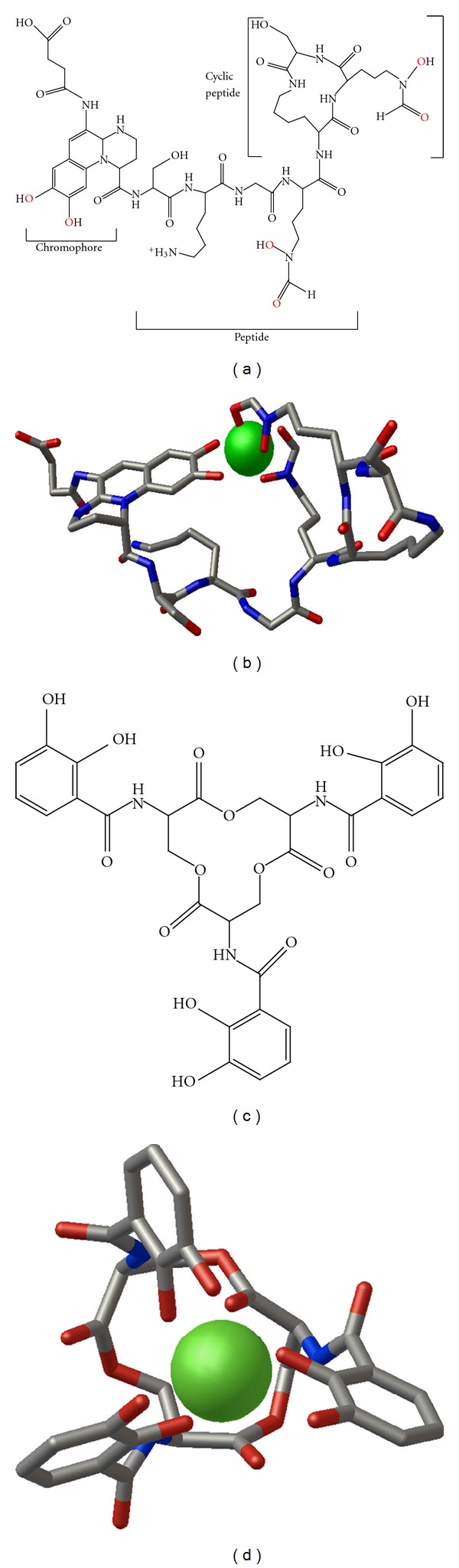
Molecular structures of ferric bacterial siderophores used in this study. (a) Chemical structure of a group I pyoverdine from bacterial species *Pseudomonas fluorescens* (strain ATCC 13525) illustrating its derivatized peptide structure. The pyoverdine amino acid sequence is Ser*-Lys-Gly-FHO-cyclic [Lys-FHO-Ser*], where Ser* is D-serine and FHO is N-formyl-N-hydroxy-ornithine. Oxygens in red represent atoms that coordinate iron in the 3D structures. (b) Ferric pyoverdine structure taken from the 3D structure of a pyoverdine-Fe transporter protein (FpvA) in complex with pyoverdine (ATCC  13525) and Fe (PDB  2W78). Only the pyoverdine and iron (green) atoms are shown. Both hydroxyl groups on the chromophore and FHO side chain oxygens form interactions with iron. (c) Chemical structure of enterobactin. (d) Ferric enterobactin structure isolated from the 3D structure of a mutant of NGAL in complex with ferric enterobactin (PDB  3CMP). Illustrations for (b) and (d) were made using Python Molecular Viewer 1.5.4. Each ligand is shown as a stick figure with coloring by atom type. Iron atoms are shown in green space-filling representation.

Cystic fibrosis (CF) is an inherited disease resulting in the formation of thick, sticky mucus in the lungs and digestive pathways [1]. CF lungs are breeding grounds for a variety of bacterial pathogens. *Pseudomonas aeruginosa* is the most common bacterial isolate that persists in CF lungs by evading host defenses and clearance mechanisms, contributing to declining lung function [1, 2]. In order to grow and thrive, bacteria need iron as a cofactor for many metabolic enzymes. However, in the case of infection, iron is not freely available in the host and is predominantly found in iron-binding proteins such as transferrin, lactoferrin, and ferritin [3]. Thus, due to the low bioavailability of free iron, bacteria produce iron-chelating small molecules known as siderophores that are secreted in apo-form and imported as ferric complexes [1, 4]. *P. aeruginosa *produces two siderophores, pyoverdine (Figures 1(a), and 1(b)) and pyochelin, that are found to be important for biofilm development and bacterial virulence [4].

As a host defense strategy, mammals have evolved strategies to limit bacterial growth by producing antibacterial iron-depleting innate immune defense proteins, such as neutrophil-gelatinase-associated lipocalin (NGAL), that specifically scavenge bacterial ferric and apo-siderophores, therefore preventing bacteria from establishing infection [5, 6]. NGAL is a 25 kDa protein also known as siderocalin, lipocalin 2, uterocalin, and 24p3, that exhibits antibacterial activity [6]. NGAL expression is upregulated following bacterial infection in an NF*κ*B-TLR4-dependent manner [7]. NGAL-deficient mice are more susceptible to bacterial infections [8] and NGAL is required for pulmonary host defense against *Klebsiella pneumoniae* [7]. Lipocalins such as NGAL share a common tertiary structure, the so-called “lipocalin fold,” characterized by an eight-strand antiparallel *β*-barrel with a calyx that contains the ligand-binding site. NGAL is expressed and secreted upon stimulation by many cells and also acts as a growth and differentiation factor in various tissues, mainly in the kidney [9]. One study has shown that NGAL levels are highly elevated in CF serum and bronchoalveolar lavage fluid (BALF) [10]. In spite of the abundance of NGAL in serum and BALF of CF patients, *P. aeruginosa* infections persist for many years.

Pathogenic bacteria evolved under the pressure of iron limitation to survive and evade host immune systems by producing structurally modified siderophores that do not bind to NGAL [11]. Pathogenic Gram-negative bacteria harbor the *iroA* gene cluster that mediates bacterial evasion of NGAL [12, 13]. For example, *Salmonella typhimurium*, a Gram-negative bacterium, produces a glycosylated enterobactin known as salmochelin [11, 14]. No homologs of the *iroA* gene cluster were found in the published *Pseudomonas* genomes, although *P. aeruginosa* produces three types of pyoverdines [1]. Other pathogenic bacteria also produce structurally distinct siderophores that evade NGAL recognition. For example, *Bacillus anthracis*, a Gram-positive bacterium, produces a siderophore known as petrobactin that does not bind to NGAL [15]; *Klebsiella pneumonia*, a Gram-negative bacterium, produces a siderophore known as yersiniabactin [16] that also does not bind to NGAL and thereby evades the host's antibacterial iron-depleting defense. Further, *Vibrio cholerae* produces vibriobactin, a catecholate siderophore that evades NGAL recognition by unique iron coordination in the ferric vibriobactin form [17].

It is not known if pyoverdine, the major siderophore produced by *P. aeruginosa*, evades this innate defense mechanism and whether this contributes to inability of CF patients to clear this bacterium from their lungs. Therefore, the aim of this work was to determine if pyoverdine binds to the antibacterial protein NGAL. We hypothesized that NGAL does not bind pyoverdine, allowing *P. aeruginosa* to establish infections and persist in CF lungs. Hence, this iron-depleting innate host defense mechanism may be dysfunctional and impaired in CF. In this paper we employed a fluorescence-based assay to characterize the binding of pyoverdine to human NGAL and molecular modeling of NGAL pyoverdine complexes to predict possible protein-ligand interactions. Here we report that neither apo- nor ferric pyoverdine bind to NGAL, results that are further supported by computational predictions indicating lack of interaction of pyoverdine with NGAL at its conventional ligand-binding site. We conclude that pyoverdine is a stealth siderophore that evades NGAL recognition, thereby allowing *P. aeruginosa* to establish infections.

## 2. Materials and Methods

### 2.1. Reagents

Recombinant human NGAL (rhNGAL, catalog no. 1757-LC) produced in a mammalian system rather than a bacterial source was purchased from R&D Systems (Minneapolis, MN, USA). Highly purified and structurally defined pyoverdines were obtained from Sigma-Aldrich (St Louis, MO, USA) in apo-form, that is, aferric pyoverdine (catalog no. P8124), and iron-bound ferric pyoverdine (catalog no. P8374). Enterobactin (catalog no. E3910), iron standard (catalog no. 16596), 2,3-dihydroxybenzoic acid (2,3-DHBA, catalog no. 0926 KB), and sterile bovine serum albumin (BSA, endotoxin free tissue culture grade, CAS 9048468) were purchased from Sigma-Aldrich. 

### 2.2. Tryptophan Fluorescence Quenching

Fluorescence measurements were made using a SpectraMax microtiter plate reader by Molecular Devices. Human NGAL contains two tryptophan residues: W31 located at the base of the hydrophobic calyx and W79 located inside the calyx (i.e., the ligand-binding site). If pyoverdine binds to NGAL it will cause quenching of the intrinsic tryptophan fluorescence. Therefore, we employed an established tryptophan fluorescence-quenching assay [13, 15] to determine the binding of pyoverdine to NGAL. Since 2,3-DHBA is the core functional moiety in pyoverdine that binds iron, the fluorescence assay was first optimized by testing ferric iron and 2,3-DHBA complexes diluted in 0.1 M Tris, pH 8.0, as the ligand for potentially quenching intrinsic tryptophan fluorescence in rhNGAL and in sterile BSA. Enterobactin (ENT), the major siderophore from *E. coli* and other enteric bacteria, was used as a positive control. The enterobactin stock solution was dissolved in DMSO, whereas apo-pyoverdine (PVD) and ferric pyoverdine (PVD-Fe) stocks were dissolved in sterile water; then, serial dilutions of the siderophores at 100, 50, 25, and 12.5 *μ*M were made in an assay buffer (50 mM Tris, 10 mM CaCl_2_, 150 mM NaCl pH 7.5). Fifty *μ*L of each dilution was then transferred into triplicate wells (F16 Black Maxisorp Plates from Nunc). Fifty *μ*L of rhNGAL was added into each well at a final concentration of 2 *μ*M and incubated for 30 min at room temperature. Recombinant hNGAL alone was incubated with assay buffer without any siderophore and used to measure intrinsic tryptophan fluorescence. The plate was then read at excitation and emission wavelengths of 280 and 340 nm, respectively, in the endpoint mode using a microplate reader with fluorescence detection. Results are presented as the percent of tryptophan fluorescence remaining compared to rhNGAL alone.

### 2.3. Molecular Modeling 

#### 2.3.1. Protein File Selection

Among 18 published crystal structures of NGAL, we selected two human NGAL structures solved by Strong and coworkers [18, 19] to serve as the basis for the molecular modeling study files 1L6M and 1QQS from the Protein Data Bank (PDB) [20]. For simplicity, we refer to the 1L6M structure as the NGAL “trimer” and the 1QQS structure as the NGAL “monomer” in this paper. Both structures were solved to 2.4 Å resolution, the highest resolution solved to date for an NGAL structure bound to enterobactin, and in the same space group (P4_1_2_1_2). The two structures are virtually identical in terms of conformation even though differences were observed in crystal packing in spite of matching space groups. Three monomers were observed in the 1L6M structure while the 1QQS structure was a homodimer with only one monomer. Moreover, NGAL has been found in a 25 kD monomeric and 45 kD dimeric biological forms, as well as a 135 kD heterodimer with MMP9 [21–23]. Due to the numerous structural similarities, minor distinctions in crystal packing, and the availability of different quaternary forms of NGAL, the 1L6M and 1QQS structures were selected as interesting models for predicting different types of interactions that pyoverdine might be able to make with NGAL and for proposing structure-activity relationships.

Another important structural feature relevant to this study is the presence and location of tryptophan residues in the NGAL structures. Biologically, human NGAL has two tryptophan residues W31 and W79. However, coordinates for the NGAL monomer (1QQS) contained a W79A where the side chain of W79 was disordered in the structure and modeled as an alanine. Otherwise, all other tryptophans present in the crystal structures were used for molecular modeling.

#### 2.3.2. Protein File Preparation

Coordinates for the NGAL trimer were extracted from PDB file 1L6M. All heteroatoms including water molecules and definitions for symmetry were excluded from the file. Coordinates for the monomeric form of NGAL were extracted from PDB file 1QQS. All water atoms were excluded from the file. Covalent attachment of the sugars N-acetyl-D-glucosamine and *α*-D-mannose and the lipid decanoic acid to the monomer protein were retained. Polar hydrogens were added to each protein and each set of modified PDB coordinates was converted to PDBQT format using AutoDock Tools [24]. PDBQT format includes information about partial atomic charges and atom types in addition to the otherwise conventional PDB format.

#### 2.3.3. Ligand File Preparation

Coordinates for ferric pyoverdine (PVD-Fe) were extracted from PDB file 2W78, which describes a complex between iron-bound PVD-ATCC13535 and its transporter protein from *P. aeruginosa*. The specific form of pyoverdine used has a chemical formula of C_17_H_17_N_3_O_6_. All other heteroatoms including the sulfate counterion were excluded. The iron atom was excluded from the working ferric pyoverdine file to generate the analogous coordinates for pyoverdine without iron bound (i.e., apo-pyoverdine). Coordinates for the ferric enterobactin control were extracted from PDB file 3CMP. Other heteroatoms present in the 3CMP file including the sodium and sulfate counterions were excluded. Connectivity definitions between atoms for each ligand file were included. Modified PDB files were converted to PDBQT format using AutoDock Tools.

#### 2.3.4. Docking

AutoDock Vina [24] was used to dock the ligand into the receptor protein. The protein was kept rigid in all experiments. Prior to the docking experiment, a grid box was selected in AutoDock Tools to encompass all atoms within either the modified NGAL monomer or trimer structure. The search space for AutoDock Vina was defined to be the dimensions of the 3D grid box. Output files were visualized using Python Molecular Viewer (PMV 1.5.4; [27]). 

## 3. Results

### 3.1. Pyoverdine Does Not Bind to NGAL

NGAL is an antibacterial innate defense protein that limits bacterial growth by binding to and sequestering siderophores [6]. We hypothesized that NGAL does not bind pyoverdine, allowing *P. aeruginosa* to persist in CF lungs. To test this hypothesis, we measured the direct binding of pyoverdine (Figure 1) to rhNGAL using the tryptophan fluorescence-quenching method [13]. Human NGAL contains two tryptophan residues: W31, which is located at the base of the hydrophobic calyx, and W79, located at the ligand-binding site. If pyoverdine binds to NGAL it would be expected to cause the quenching of the intrinsic fluorescence of tryptophan.

**Figure 2:**
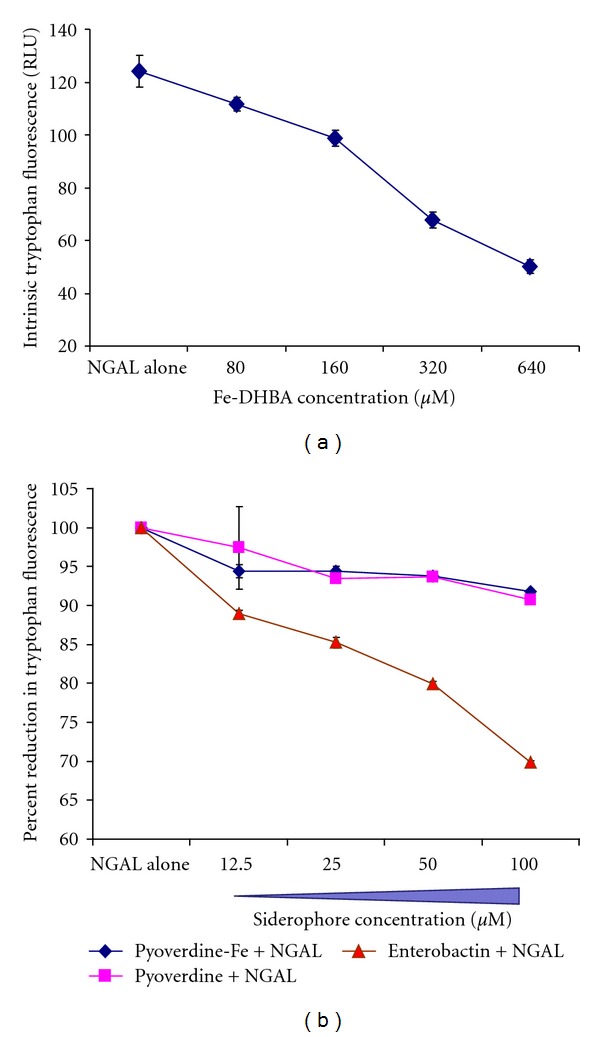
Pyoverdine does not bind to NGAL. The binding of pyoverdine and enterobactin to NGAL was measured by a tryptophan fluorescence-quenching method [13, 15]. Siderophore dilutions were preincubated with recombinant human NGAL (2 *μ*M) for 30 min prior to reading with a plate reader. (a) Optimization of intrinsic tryptophan fluorescence quenching in rhNGAL by Ferric-DHBA complexes in a dose-dependent manner. (b) Percent of reduction in tryptophan fluorescence upon enterobactin binding to NGAL compared to apo-pyoverdine and ferric pyoverdine. Error bars (some smaller than the symbol) represent ±SD from the average of triplicate readouts at each point.

The quenching of the intrinsic tryptophan fluorescence of rhNGAL in a dose-dependent manner was first optimized using iron complexed to 2,3-dihydroxybenzoic acid (Fe-DHBA) (Figure 2(a)). As expected, increasing concentrations of Fe-DHBA led to increased quenching, suggesting binding of the siderophore core iron-binding structure to NGAL. As another positive control, enterobactin induced a response similar to that of Fe-DHBA. Ferric enterobactin-NGAL complexes showed a fluorescence decrease as the siderophore concentration increased, indicating fluorescence quenching and binding of the ligand to NGAL (Figure 2(b)). In contrast to enterobactin, neither apo-pyoverdine nor ferric pyoverdine reduced the intrinsic tryptophan fluorescence of rhNGAL significantly (Figure 2(b)). Tryptophan fluorescence was only diminished by less than 10% of the NGAL baseline fluorescence even when a high dose of pyoverdine was used, suggesting that pyoverdine does not bind to NGAL even when complexed with iron which is known to increase the affinity of siderophore binding to NGAL [12]. The data suggest that NGAL does not bind pyoverdine. Thus, NGAL does not exert an antibacterial function against *P. aeruginosa* and, therefore, pyoverdine is a stealth siderophore.

### 3.2. Pyoverdine-NGAL Interactions Predicted from Molecular Modeling

#### 3.2.1. Apo-Pyoverdine Docking to NGAL

**Figure 3:**
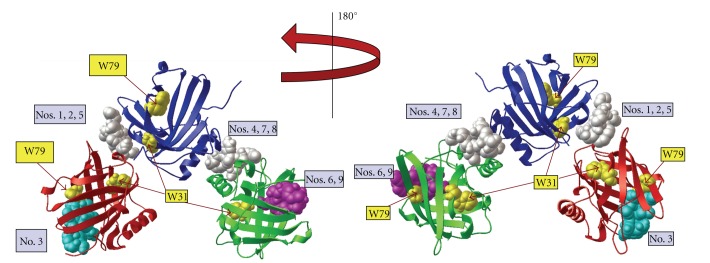
Apo-pyoverdine does bind to NGAL. Docking of apo-pyoverdine (without iron) into the NGAL trimer crystal structure (PDB 1L6M) using AutoDock Vina. NGAL protein in red, blue, and green ribbon diagrams with nine pyoverdine docking modes shown in space-filling representation. Here, each of the binding regions is displayed on only one representative pyoverdine molecule (turquoise, white, or magenta). Tryptophan residues W31 and W79 are highlighted in yellow. The model was rotated 180° for display in the view at right. The binding affinity for each docking mode is listed Table 1.

Docking of apo-pyoverdine (Figure 1(a)) to the NGAL trimer predicted nine different docking sites or modes (Figure 3(a)). Six of the nine modes (nos. 1, 2, 4, 5, 7, 8, Figure 3) clustered at an interface between subunits within the NGAL trimer. The remaining binding modes (nos. 3, 6, 9, Figure 3) were at the periphery of a subunit near the NGAL calyx without being fully buried within the hydrophobic binding pocket. When apo-pyoverdine docked at the periphery of a subunit, the ligand was also near a tryptophan residue. Docking modes 6 and 9 for apo-pyoverdine were in proximity to both W31 and W79 as best seen in the 180° rotational view (Figure 3, right). In docking mode 3, apo-pyoverdine is only in proximity to W79. All nine of the docking modes for the apo-pyoverdine ligand localized to the outer surface of the NGAL monomer (data not shown). In eight of the nine docking modes, apo-pyoverdine docked near the opening of the calyx but not within the ligand-binding pocket.

#### 3.2.2. Ferric Pyoverdine Docking to NGAL

**Figure 4:**
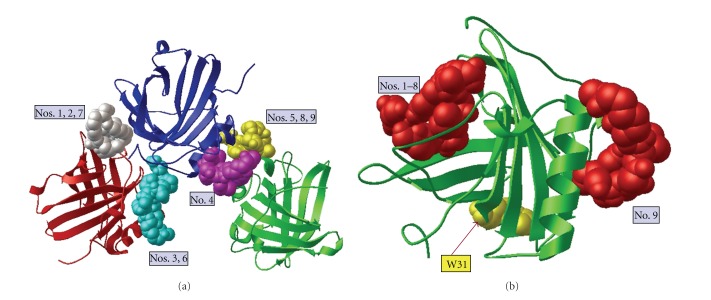
Ferric pyoverdine does not dock inside the NGAL calyx. (a) Docking of ferric pyoverdine (bound to iron) into the NGAL trimer crystal structure (PDB 1L6M) using AutoDock Vina. NGAL protein in red, blue, and green ribbon diagrams with nine positions of docked pyoverdine shown. For this illustration only one pyoverdine molecule is displayed in each of the predicted binding positions on the rigid NGAL. All simulated docking modes were on the surface and between the units of NGAL but not near or in the ligand-binding site. The binding affinity for each docking mode is listed in Table 1. (b) Ferric pyoverdine docking into the NGAL monomer crystal structure (PDB 1QQS) using AutoDock Vina. Modes 1–8 docked near the opening of the NGAL calyx without fully fitting in the hydrophobic pocket, while mode 9 docked on the opposite surface. The binding affinity for each docking mode is listed Table 1.

As with the apo-pyoverdine structures, AutoDock Vina predicted nine positions in which ferric pyoverdine could dock into the NGAL trimer; however, none of the ferric pyoverdine molecules were predicted to bind near or inside NGAL's ligand binding pocket (Figure 4(a)). Each ferric pyoverdine docked between the NGAL subunits. Further, AutoDock Vina predicted nine positions in which ferric pyoverdine could dock onto the NGAL monomer structure. In each case, however, the position of the docked ligand was at the surface of the protein and not near or inside the calyx (Figure 4(b)). This computational data strongly suggests that ferric pyoverdine does not bind to NGAL, which is consistent with the experimental results.

#### 3.2.3. Enterobactin Docking to NGAL

**Figure 5:**
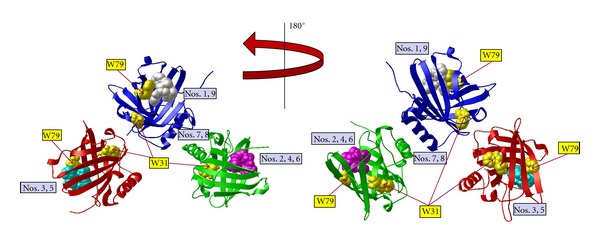
Enterobactin binds to NGAL. The docking of ferric enterobactin into the NGAL crystal structure (PDB 1L6M) using AutoDock Vina was performed as a positive control to validate the pyoverdine docking simulation. NGAL crystal structure with each monomer presented in ribbon diagram colored red, blue, and green, plus ferric enterobactin. AutoDock Vina found nine positions in which ferric enterobactin docked, seven of which were in one of the calyx of one of the monomers, that is, the ligand-binding site. The two remaining positions, modes 7 and 8, are between the blue and green monomers (enterobactin is not shown in modes 7 and 8, for clarity). For this illustration only one ferric enterobactin molecule is displayed in each of the predicted binding positions on the rigid NGAL. Tryptophan residues W31 and W79 are highlighted in yellow. The docking of ferric enterobactin to NGAL view was rotated 180° for display in the view at right. The binding affinity for each docking mode is listed in Table 1.

**Figure 6:**
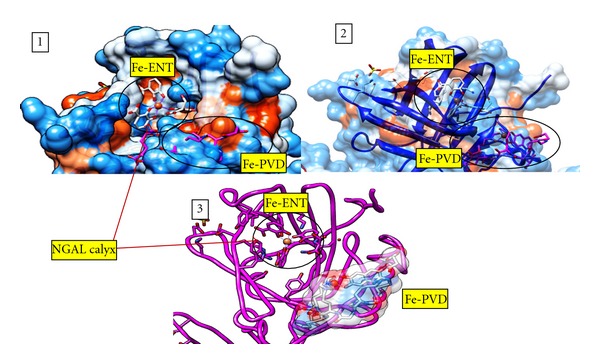
Ferric pyoverdine superimposed on ferric Enterobactin docked into NGAL. The docking of ferric pyoverdine to NGAL is superimposed on ferric enterobactin bound to NGAL. (1) NGAL molecular surface representation with Fe-ENT bound in the hydrophobic calyx where Fe-PVD docking site is buried under the surface. In the surface representation, blue indicates a hydrophobic region, while red indicated a hydrophilic region. Fe-ENT and Fe-PVD are shown as stick figures. (2) NGAL molecular surface representation with 50% transparency. (3) NGAL backbone (fuchsia) with the Fe-PVD surface shown in 50% transparency. This figure was generated using the UCSF Chimera package [25, 26].

Enterobactin, the major siderophore in *E. coli* and many other pathogenic enteric bacteria, binds to NGAL with very high affinity [18, 28]. The binding and molecular docking of ferric enterobactin to NGAL is well established and extensively investigated [18]. In order to validate the docking results obtained for pyoverdine, ferric enterobactin (Figure 1(d)) was used as a positive control for computational docking to the NGAL trimer (Figure 5) and monomer (data not shown). AutoDock Vina predicted 9 positions in which ferric enterobactin could dock into the trimeric NGAL protein crystal structure PDB 1L6M. Seven different docking modes (nos. 1, 2, 3, 4, 5, 6, and 9) indicate that ferric enterobactin could dock inside the NGAL calyx as been reported in the crystal structure of ferric enterobactin-bound NGAL [18]. The remaining two docking modes (nos. 7 and 8) predicted ferric enterobactin docking away from the calyx and between the NGAL subunits (Figure 5). Overall, AutoDock Vina accurately simulated the binding of ferric enterobactin to the NGAL trimer at the ligand binding site (i.e., inside the calyx of NGAL). Further, ferric pyoverdine was superimposed on ferric enterobactin docked into NGAL calyx with a transparent surface presentation. The results reflect again the inability of ferric pyoverdine to dock into the calyx (Figure 6).

### 3.3. Predicted Binding Affinities

AutoDock Vina provides a computed binding affinity for each docking mode predicted. Binding affinity data are summarized in Table 1. The more negative the value, the tighter the predicted binding. For the apo-pyoverdine interaction with the NGAL trimer, predicted binding affinities were very similar for the nine modes, ranging from −6.4 to −6.9 kcal/mol. Predicted binding affinities for apo-pyoverdine with the NGAL monomer (−5.8 to −6.5 kcal/mol) were slightly lower in range than those for the analogous trimer complex (Table 1). Data for ferric pyoverdine interactions gave slightly tighter predicted binding than that for apo-pyoverdine interactions: predicted binding energies of −8.2 to −9.2 kcal/mol for the trimer and −7.6 to −8.9 kcal/mol for the monomer. Consistent with biological trends, iron-bound siderophores were predicted to have stronger binding interactions with NGAL monomer or trimer than their corresponding apo-forms. In all permutations, docking mode 1 represented the highest affinity conformation of the ligand interacting with the NGAL. In every case, docking mode 1 was computed to have a higher binding energy in interaction with the trimer over the monomer, although even this mode for pyoverdine gave low predicted affinity. In contrast, the tightest predicted binding affinities were computed for the ferric enterobactin interactions with the NGAL trimer (−9.7 to −11.8 kcal/mol). While all of the predicted binding affinities are moderate in scale, none of the pyoverdine values are comparable to the range predicted for ferric enterobactin binding with NGAL.

## 4. Discussion

*P. aeruginosa* is an opportunistic pathogen that causes severe infection not only in CF patients but also in critically ill patients in intensive care units where it can cause ventilator-associated infections. It is a major nosocomial pathogen associated with significant morbidity and mortality (40–60%) in immunocompromised patients and in certain hospital units like burn and palliative care [29]. *P. aeruginosa* possesses several virulence factors, one of which is pyoverdine, the major siderophore that sequesters iron from the host to facilitate bacterial growth [30, 31]. Pyoverdine is a virulence factor required for establishing infection and biofilm formation [32]. In response to infection, the host secretes NGAL that sequesters bacterial siderophores thereby preventing bacteria from establishing infection [6]. 

The role of NGAL in innate immunity is well established and although NGAL-deficient mice develop normally, they are more susceptible to bacterial infections. NGAL is required for pulmonary host defense against *Klebsiella pneumonaie* [7], indicating that NGAL is an important mediator in innate host defense [8]. However, not all bacterial siderophores are recognized by NGAL and some pathogenic bacteria modify their siderophore structure to evade NGAL recognition [11–13, 16, 17]. It has been shown that salmochelin, a major siderophore in pathogenic *Salmonella*, is a glucosylated form of enterobactin [11, 12, 33]. The glycosylation renders the molecule bulky such that it would not fit within the NGAL calyx, thus evading innate immune defense. Pyoverdine, a catecholate-based molecule, is not a glycosylated siderophore. *P. aeruginosa* does not contain the *iroA* gene cluster responsible for the glycosylation of catecholate-based siderophores that do not bind to NGAL [1, 3]. Compared to other dihydroxybenzoic-acid-(DHBA-) based catecholates that bind to NGAL such as enterobactin, pyoverdine is a structurally bulky molecule due to its dihydroxyquinoline chromophore [1]. We postulated that pyoverdine is a stealth siderophore that does not bind to NGAL. Here we show that pyoverdine evades NGAL recognition and therefore likely facilitates iron acquisition by *P. aeruginosa* which supports establishing infection. 

Pyoverdine is uniquely produced by *Pseudomonas* bacteria. Using the tryptophan fluorescence-quenching method, we found that pyoverdine did not bind to NGAL, neither in the apo-form nor when complexed with iron. In contrast, enterobactin bound to NGAL causing strong tryptophan quenching. Using molecular docking we showed that pyoverdine did not dock into the calyx of NGAL while identical approaches indicate that enterobactin docks to NGAL with high affinity. We conclude that pyoverdine is a virulence factor and a stealth siderophore which allows *P. aeruginosa* to establish infection. 

Lipocalins act as carrier proteins for a variety of ligands including small-molecule drugs [34, 35]. Other members of the lipocalin family have also been shown to act as siderocalins, that is, binding and sequestering bacterial siderophores [36, 37]. Tear lipocalin, also called LCN1, has been shown to sequester enterobactin but not pyoverdine which lends strong support to our results [38, 39]. Unlike NGAL that preferably binds catecholate-based siderophores [28], tear lipocalin can bind wide range of bacterial and fungal siderophores including catecholate-based and hydroxamate-based siderophores [39]. In contrast to NGAL and tear lipocalin, galline Ex-FABP, a lipocalin expressed in chicken serum and egg white, has been reported to sequester both glucosylated enterobactin and pyoverdine. It is also a sensor for lysophosphatidic acid and is reported to have dual ligand specificities [40]. The recently published crystal structure of galline Ex-FABP explained the dual ligand specificity due its expanded multichambered cavity [40]. NGAL is reported to be a rigid and quite stable lipocalin with a smaller calyx that does not undergo conformational changes upon ligand binding [18, 19] which explains why pyoverdine binds to Ex-FABP but not to NGAL. Similar to NGAL, galline Ex-FABP was shown to exert strong bacteriostatic activity against *E. coli* and *B. subtilis* but not against *Pseudomonas aeruginosa* due to low affinity of interaction between Ex-FABP and ferric pyoverdine [40]. We also performed similar bacterial growth inhibition assay with NGAL and *P. aeruginosa* and found only 10% reduction in bacterial growth (data not shown); therefore we concluded that NGAL is not bacteriostatic for *P. aeruginosa*.

*P. aeruginosa* produces another siderophore called pyochelin which is a small chatecholate-based molecule with iron-binding affinity [32]. Recently, Strong and coworkers crystallized NGAL bound to pyochelin (PDB 3U03, deposited at the RCSB protein data bank) and the crystal structure clearly shows that pyochelin docks inside the calyx of NGAL but without binding avidly. However, pyochelin is not essential for establishing infection since a *P. aeruginosa *mutant that lacks pyochelin successfully established infection and formed a biofilm [4]. Chronic infections caused by *P. aeruginosa* as seen in CF are associated with biofilms that protect the bacteria against the host killing mechanisms as well as increase resistance to antibiotics [4, 30]. In established biofilms of CF airways, *P. aeruginosa* thrives and downregulates pyoverdine since iron is abundant in the airway surface liquid due to chronic inflammation [41, 42]. Therefore, pyoverdine is important for establishing infections but not for well-established infections like that in biofilms. 

In summary, we show that pyoverdine, the major siderophore in *P. aeruginosa*, is a stealth siderophore that evades NGAL recognition.

## Figures and Tables

**Table 1:** Predicted binding affinities (kcal/mol).

Docking mode	Apo pyoverdine		Ferric pyoverdine		Ferric enterobactin
	Trimer	Monomer	Trimer	Monomer	Trimer
1	−6.9	−6.5	−9.2	−8.9	−11.8
2	−6.8	−6.4	−9.1	−8.4	−11.3
3	−6.8	−6.3	−8.7	−8.3	−10.9
4	−6.7	−6.3	−8.6	−8.2	−10.9
5	−6.6	−6.3	−8.6	−8.2	−10.8
6	−6.6	−6.0	−8.3	−8.1	−10.5
7	−6.5	−5.9	−8.3	−8.0	−10.4
8	−6.5	−5.8	−8.3	−7.9	−9.8
9	−6.4	−5.8	−8.2	−7.6	−9.7

## References

[1] Visca P, Imperi F, Lamont IL (2007). Pyoverdine siderophores: from biogenesis to biosignificance. *Trends in Microbiology*.

[2] Cigana C, Curcuru L, Leone MR (2009). *Pseudomonas aeruginosa* exploits lipid a and muropeptides modification as a strategy to lower innate immunity during cystic fibrosis lung infection. *PLoS ONE*.

[3] Smith KD (2007). Iron metabolism at the host pathogen interface: lipocalin 2 and the pathogen-associated iroA gene cluster. *International Journal of Biochemistry and Cell Biology*.

[4] Lamont IL, Konings AF, Reid DW (2009). Iron acquisition by *Pseudomonas aeruginosa* in the lungs of patients with cystic fibrosis. *BioMetals*.

[5] Saiga H, Nishimura J, Kuwata H (2008). Lipocalin 2-dependent inhibition of mycobacterial growth in alveolar epithelium. *Journal of Immunology*.

[6] Flo TH, Smith KD, Sato S (2004). Lipocalin 2 mediates an innate immune response to bacterial infection by sequestrating iron. *Nature*.

[7] Chan YR, Liu JS, Pociask DA (2009). Lipocalin 2 is required for pulmonary host defense against Klebsiella infection. *Journal of Immunology*.

[8] Berger T, Togawa A, Duncan GS (2006). Lipocalin 2-deficient mice exhibit increased sensitivity to *Escherichia coli* infection but not to ischemia-reperfusion injury. *Proceedings of the National Academy of Sciences of the United States of America*.

[9] Schmidt-Ott KM, Mori K, Jau YL (2007). Dual action of neutrophil gelatinase-associated lipocalin. *Journal of the American Society of Nephrology*.

[10] Eichler I, Nilsson M, Rath R, Enander I, Venge P, Koller DY (1999). Human neutrophil lipocalin, a highly specific marker for acute exacerbation in cystic fibrosis. *European Respiratory Journal*.

[11] Müller SI, Valdebenito M, Hantke K (2009). Salmochelin, the long-overlooked catecholate siderophore of Salmonella. *BioMetals*.

[12] Fischbach MA, Lin H, Liu DR, Walsh CT (2006). How pathogenic bacteria evade mammalian sabotage in the battle for iron. *Nature Chemical Biology*.

[13] Fischbach MA, Lin H, Zhou L (2006). The pathogen-associated iroA gene cluster mediates bacterial evasion of lipocalin 2. *Proceedings of the National Academy of Sciences of the United States of America*.

[14] Lin H, Fischbach MA, Liu DR, Walsh CT (2005). In vitro characterization of salmochelin and enterobactin trilactone hydrolases IroD, IroE, and Fes. *Journal of the American Chemical Society*.

[15] Abergel RJ, Wilson MK, Arceneaux JEL (2006). Anthrax pathogen evades the mammalian immune system through stealth siderophore production. *Proceedings of the National Academy of Sciences of the United States of America*.

[16] Bachman MA, Oyler JE, Burns SH (2011). Klebsiella pneumoniae yersiniabactin promotes respiratory tract infection through evasion of lipocalin 2. *Infection and Immunity*.

[17] Li N, Zhang C, Li B (2012). Unique iron coordination in iron-chelating molecule vibriobactin helps Vibrio cholerae evade mammalian siderocalin-mediated immune response. *The Journal of Biological Chemistry*.

[18] Goetz DH, Holmes MA, Borregaard N, Bluhm ME, Raymond KN, Strong RK (2002). The neutrophil lipocalin NGAL is a bacteriostatic agent that interferes with siderophore-mediated iron acquisition. *Molecular Cell*.

[19] Goetz DH, Willie ST, Armen RS, Bratt T, Borregaard N, Strong RK (2000). Ligand preference inferred from the structure of neutrophil gelatinase associated lipocalin. *Biochemistry*.

[20] Berman HM, Westbrook J, Feng Z (2000). The protein data bank. *Nucleic Acids Research*.

[21] Mori K, Lee HT, Rapoport D (2005). Endocytic delivery of lipocalin-siderophore-iron complex rescues the kidney from ischemia-reperfusion injury. *The Journal of Clinical Investigation*.

[22] Cai L, Rubin J, Han W, Venge P, Xu S (2010). The origin of multiple molecular forms in urine of HNL/NGAL. *Clinical Journal of the American Society of Nephrology*.

[23] Hu L, Hittelman W, Lu T (2009). NGAL decreases E-cadherin-mediated cellcell adhesion and increases cell motility and invasion through Rac1 in colon carcinoma cells. *Laboratory Investigation*.

[24] Trott O, Olson AJ (2010). Software news and update AutoDock Vina: improving the speed and accuracy of docking with a new scoring function, efficient optimization, and multithreading. *Journal of Computational Chemistry*.

[26] Pettersen EF, Goddard TD, Huang CC (2004). UCSF Chimera—a visualization system for exploratory research and analysis. *Journal of Computational Chemistry*.

[27] Sanner MF (1999). Python: a programming language for software integration and development. *Journal of Molecular Graphics and Modelling*.

[28] Holmes MA, Paulsene W, Jide X, Ratledge C, Strong RK (2005). Siderocalin (Lcn 2) also binds carboxymycobactins, potentially defending against mycobacterial infections through iron sequestration. *Structure*.

[29] Jones RN (2010). Microbial etiologies of Hospital-acquired bacterial pneumonia and ventilator-associated bacterial pneumonia. *Clinical Infectious Diseases*.

[30] Goldberg JB (2010). Why is *Pseudomonas aeruginosa* a pathogen?. *F1000 Biology Reports*.

[31] Khalifa ABH, Moissenet D, Thien HV, Khedher M (2011). Virulence factors in *Pseudomonas aeruginosa*: mechanisms and modes of regulation. *Annales de Biologie Clinique*.

[32] Poole K, McKay GA (2003). Iron acquisition and its control in *Pseudomonas aeruginosa*: many roads lead to Rome. *Frontiers in Bioscience*.

[33] Fischbach MA, Lin H, Liu DR, Walsh CT (2005). In vitro characterization of IroB, a pathogen-associated C-glycosyltransferase. *Proceedings of the National Academy of Sciences of the United States of America*.

[34] Xu S, Venge P (2000). Lipocalins as biochemical markers of disease. *Biochimica et Biophysica Acta*.

[35] Flower DR (1994). The lipocalin protein family: a role in cell regulation. *FEBS Letters*.

[36] Hoette TM, Clifton MC, Zawadzka AM, Holmes MA, Strong RK, Raymond KN (2011). Immune interference in Mycobacterium tuberculosis intracellular iron acquisition through siderocalin recognition of carboxymycobactins. *ACS Chemical Biology*.

[37] Abergel RJ, Clifton MC, Pizarro JC (2008). The siderocalin/enterobactin interaction: a link between mammalian immunity and bacterial iron transport. *Journal of the American Chemical Society*.

[38] Dartt DA (2011). Tear lipocalin: structure and function. *Ocular Surface*.

[39] Fluckinger M, Haas H, Merschak P, Glasgow BJ, Redl B (2004). Human tear lipocalin exhibits antimicrobial activity by scavenging microbial siderophores. *Antimicrobial Agents and Chemotherapy*.

[40] Correnti C, Clifton MC, Abergel RJ (2011). Galline Ex-FABP is an antibacterial siderocalin and a lysophosphatidic acid sensor functioning through dual ligand specificities. *Structure*.

[41] Banin E, Vasil ML, Greenberg EP (2005). Iron and *Pseudomonas aeruginosa* biofilm formation. *Proceedings of the National Academy of Sciences of the United States of America*.

[42] Wang Y, Wilks JC, Danhorn T, Ramos I, Croal L, Newman DK (2011). Phenazine-1-carboxylic acid promotes bacterial biofilm development via ferrous iron acquisition. *Journal of Bacteriology*.

